# A Rare Case of a Large, Deceitfully Quiet Brainstem Arteriovenous Malformation Presenting Only as Dizziness

**DOI:** 10.7759/cureus.8870

**Published:** 2020-06-27

**Authors:** Swathi Rao, Fanny Giron

**Affiliations:** 1 Internal Medicine, MacNeal Hospital, Berwyn, USA

**Keywords:** arteriovenous malformation, dizziness, nystagmus, vertigo

## Abstract

Dizziness is one of the most frequent complaints encountered in the medical practice affecting 15%-20% of adults yearly, and can be challenging to assess. Most patients use dizziness as a non-specific term, and thus suffer prejudice from the physicians’ end and can be disregarded frequently. Dizziness can be a symptom of various diseases, some with sinister pathologies. We present a case of garden-variety vertigo that unfurled to be not-so-simple, emphasizing the importance of a thorough history and physical examination again even in the era of technology. A 32-year-old male patient with no past medical history presented with dizziness, later clarified as gradually progressive vertigo for two years, with unstable gait, dysarthria, and occasional diplopia. Physical examination found sustained nystagmus that changed direction with horizontal gaze, vertical nystagmus with upward gaze, dysarthria, and a wide-based ataxic gait. CT head without contrast revealed indeterminate hypodense areas in the left midbrain, pons, and cerebellar hemisphere. MRI brain identified a 2.8 x 3.4 x 4.2 cm Spetzler-Martin grade IV brainstem arteriovenous malformation (AVM) involving the left midbrain, pons, and cerebellum. Feeders were mostly from the posterior circulation, with three intranidal aneurysms, all draining into the deep venous system. The AVM was deemed inoperable, and the patient was treated with onyx embolization for two/three feeding vessel aneurysms. After treatment, the symptoms persisted, and the patient was diagnosed with major depressive disorder (MDD) six months after diagnosis, and was admitted a year later with suicidal ideation and substance use disorder. Brain AVMs are rare clinical entities that present in 0.1% of the population, mostly presenting as intracranial bleeds. When they do rarely present with isolated focal neurologic deficits, it has been attributed to a vascular steal phenomenon, hemorrhage, or a mass effect. The isolated findings of vertigo and dysarthria are highly non-specific; with such presentation, clinicians should consider etiologies under the realm of vertigo of central origin. An untreatable AVM reduces patients’ quality of life and has been linked to depression and anxiety, and thus patients may benefit from psychosocial therapy. Although preventing intracranial hemorrhage (ICH) is the primary concern with brain AVMs, the rest of the patient’s profile should not be forgotten.

## Introduction

Dizziness is one of the most frequent complaints encountered in the medical practice affecting 15%-20% of adults yearly, and it can be a challenging symptom to assess [1]. Both history and physical exam are key components to ascertain the etiology of the patient’s dizziness. Nearly 40% of patients complaining of dizziness are found to have peripheral vertigo, and 10% are found to have vertigo of central origin [2,3]. Vertigo is a subtype of dizziness defined as an illusory sensation of movement. Reaching the correct diagnosis can become puzzling for physicians because it encompasses a wide range of diagnosis starting from a benign condition to a life-threatening disease.

Peripheral vertigo is usually characterized by imbalance, nausea, vomiting, and nystagmus that can be horizontal or mixed; the presence of ear symptoms makes this diagnosis more likely. Conversely, central vertigo is associated with severe imbalance to the point where sometimes patients are unable to walk, additional neurologic manifestations may also be present, nausea and vomiting are not as pronounced, and nystagmus is typically pure vertical or torsional [4]. Here we present a singular case of vertigo, proving how important it is to have a high clinical suspicion when a patient with this symptom walks in. 

## Case presentation

**Figure 1:**
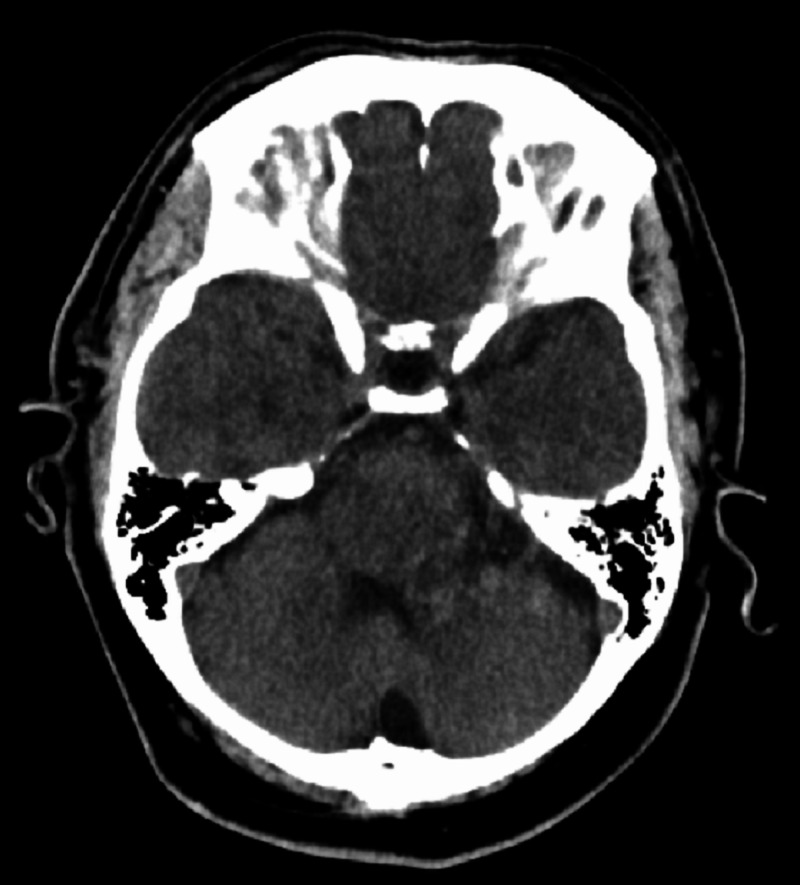
CT head without contrast, axial section

We present a rare case of an infratentorial brainstem arteriovenous malformation (AVM). A 32-year-old male patient with no other past medical history presented with dizziness which was further clarified as gradually progressive vertigo for two years, leading to unstable gait, mild dysarthria, and occasional diplopia. The symptoms were not associated with headache, nausea, vomiting, tinnitus, or hearing loss. There was no history of muscle weakness, seizures, or loss of consciousness. On thorough clinical examination, he was found to have a sustained nystagmus that changed direction with horizontal gaze and vertical nystagmus with upward gaze. He had dysarthria, a wide-based ataxic gait. A single large or multiple lesions affecting pons, midbrain, and cerebellum were suspected. Initial plain CT head (Figure 1) in emergency room revealed an indeterminate hypodense area in the left midbrain, pons, and cerebellar hemisphere.

**Figure 2:**
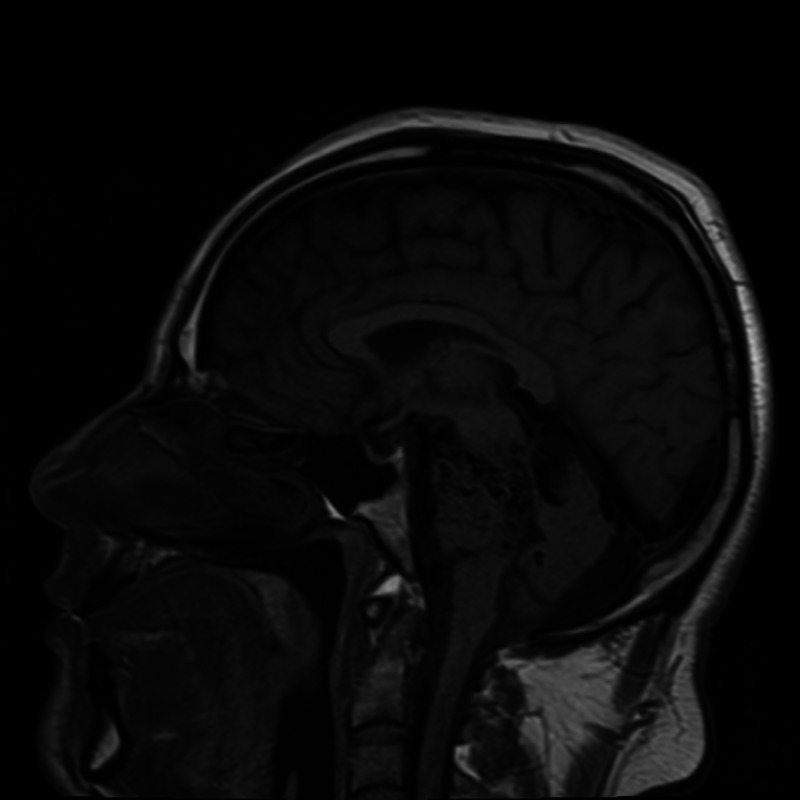
MRI head without contrast, sagittal section

**Figure 3:**
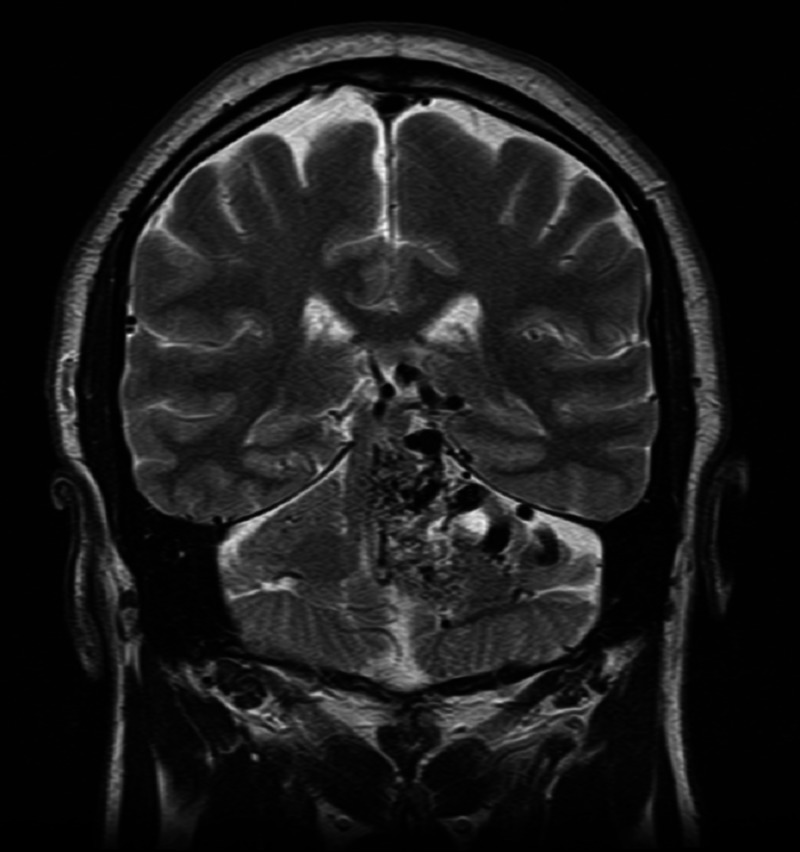
MRI head without contrast, coronal section

**Figure 4:**
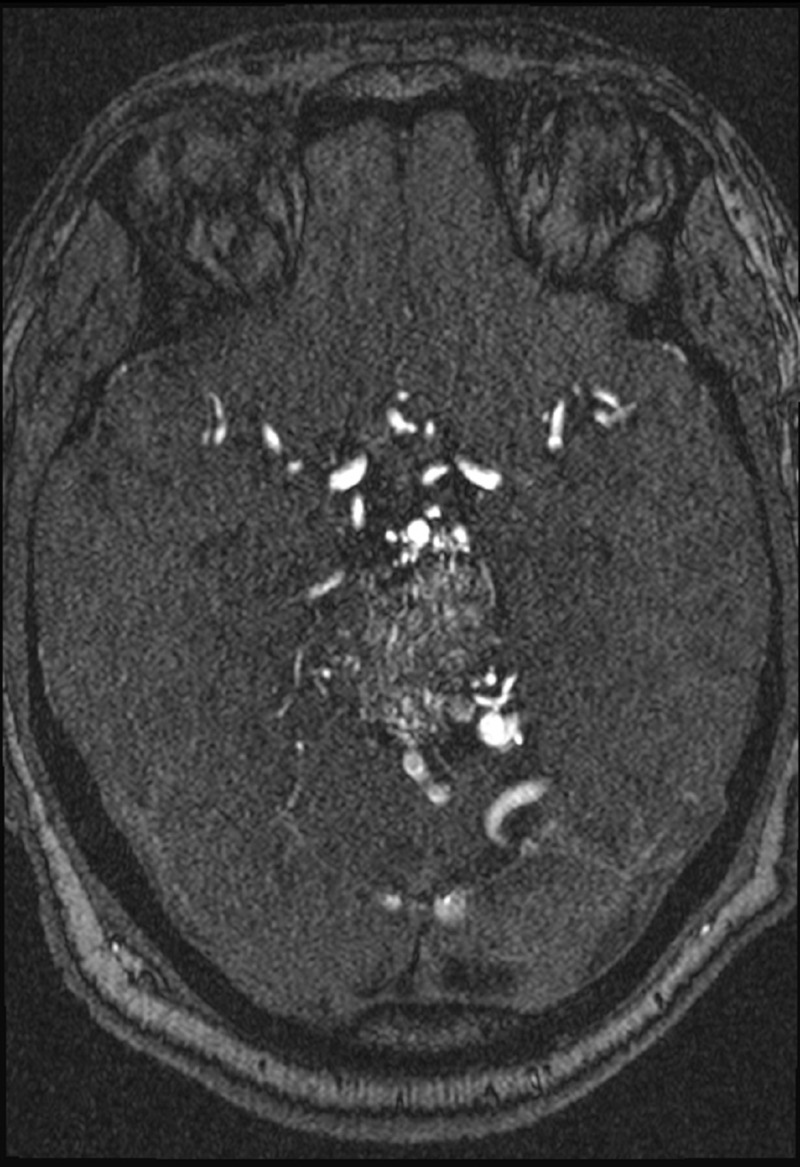
MRI head with contrast, axial section

MRI brain (Figures 2-4) identified Spetzler-Martin grade IV brainstem AVM measuring approximately 2.8 x 3.4 x 4.2 cm primarily involving the left paracentral brainstem (midbrain, pons, and cerebellum). 

**Figure 5:**
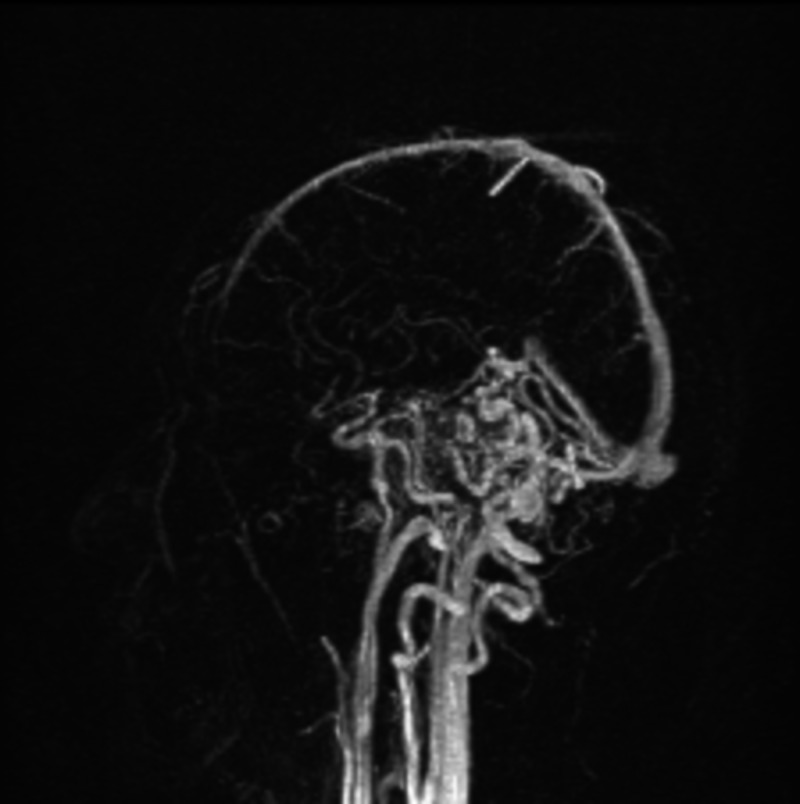
MRA head with contrast, sagittal section

**Figure 6:**
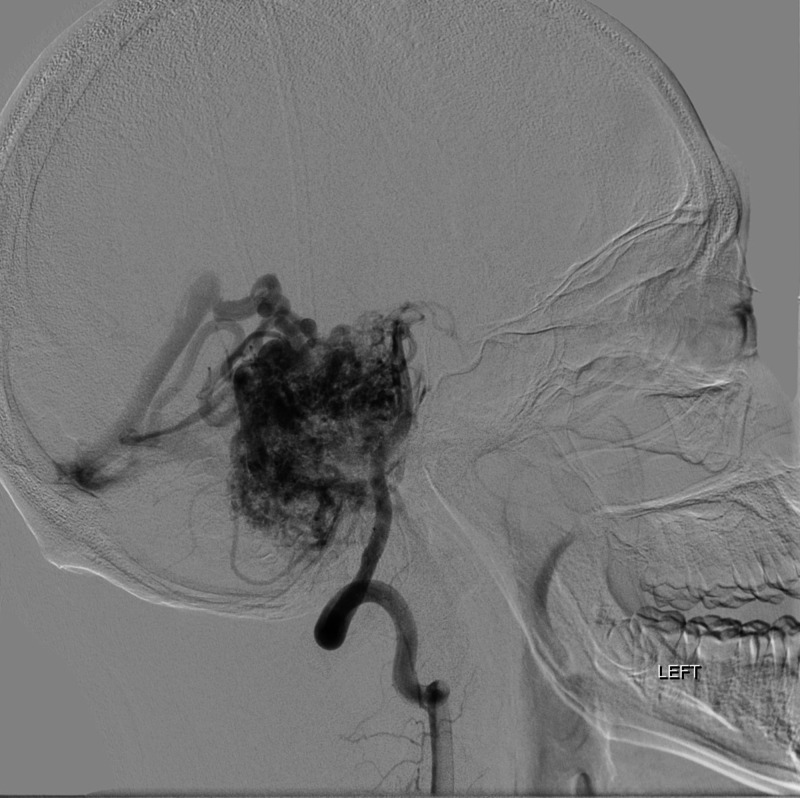
Angiography of vertebral artery, sagittal section

**Figure 7:**
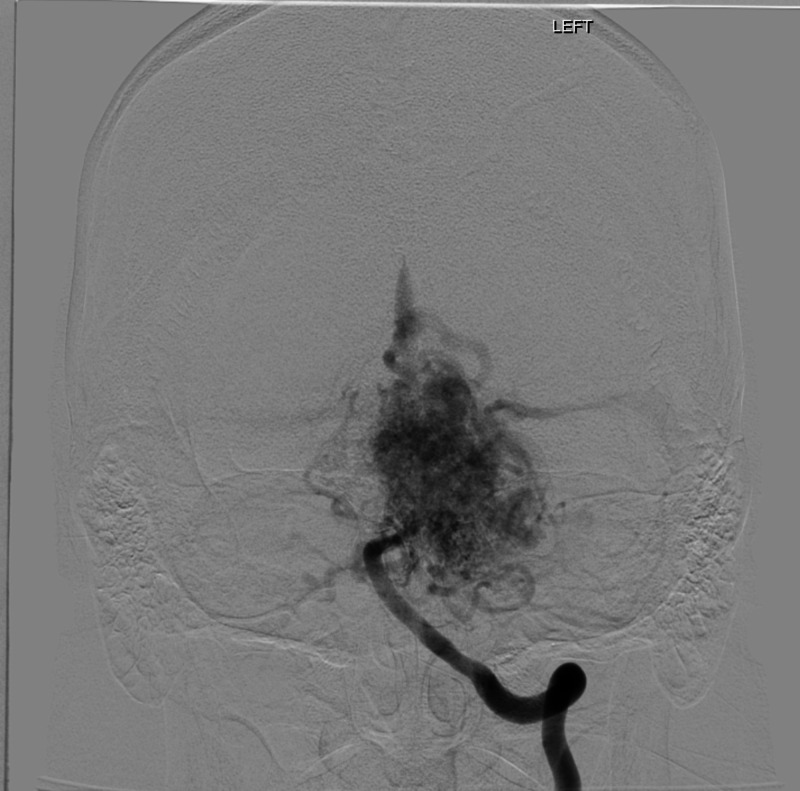
Angiography of vertebral artery, coronal section

Feeders were mostly from the posterior circulation, with three intranidal aneurysms, all draining into the deep venous system (Figures 5-7). The AVM was deemed inoperable, and the patient was treated with onyx embolization for two of the three feeding vessel aneurysms. Even following the treatment, the symptoms persisted, affecting the patient’s quality of life significantly. He was diagnosed with major depressive disorder (MDD) six months after the diagnosis and treatment and was admitted a year later with suicidal ideation and substance use disorder. 

## Discussion

This case is a rare vascular malformation presenting in an unusual location and manifestation. AVMs in the brain are rare clinical entities that present in only 0.1% of the population [5]. Of all the congenital vascular malformations, they are the most dangerous, commonly presenting with intracranial hemorrhage (ICH) (41%-79%) or seizure (11%-33%) [6-8]. Some also present with headache. When they do rarely present with isolated focal neurologic deficits, it has been attributed to a vascular steal phenomenon, hemorrhage, or a mass effect [9]. Ninety percent of AVMs are supratentorial, while the remaining occur in the posterior fossa. They are usually single and considered sporadic. Multiple lesions point to a familial disorder like hereditary hemorrhagic telangiectasia (HHT) [10,11]. Their size can vary widely, and they sometimes undergo growth or regression over time. They are angiographically seen as direct connections between arteries and veins without an intervening capillary bed and can have multiple feeder vessels. About 20%-25% develop aneurysms of the vessels, increasing the chances of an intracranial bleed [12]. Surprisingly, the size of the AVM does not seem to affect the risk of hemorrhage.

This patient presented with a posterior fossa AVM. This localization accounts for 7%-15% of all intracranial AVMs, the most common presentation for these types of AVMs are infratentorial hemorrhages (60%-86%) followed by progressive neurologic deficits and less commonly cranial nerve palsies or ataxia [13]. The isolated findings of vertigo and dysarthria without hemorrhage or seizures are highly non-specific and have a multitude of differential diagnoses; with such presentation clinicians should consider etiologies under the realm of vertigo of central origin. While approaching to vertigo, thorough history focused on duration and onset of the vertigo, and physical examination focusing on focal neurologic deficits particularly eye movements, nystagmus, cranial nerves, and cerebellar exam will help us narrow down the etiologies and identify the patients who require imaging studies. Some of the differentials considered in this case were multiple sclerosis, Wernicke’s encephalopathy, tumor or metastatic disease, and spinocerebellar ataxia. The realization of the presence of an untreatable AVM reduces the patient’s quality of life, and this finding has been linked to depression and anxiety [14-16]. It can be beneficial to refer patients to psychiatrists or therapists at the time of diagnosis or even after treatment to provide them support and assist them to build coping skills. Although preventing ICH is the primary concern with brain AVMs, rest of the patient’s profile should not be set aside.

## Conclusions

While approaching vertigo, a thorough history focused on duration and onset of the vertigo, and physical examination focusing on focal neurologic deficits, eye movements, nystagmus, cranial nerves, and cerebellar exam is pivotal. An untreatable AVM reduces patients’ quality of life and has been linked to depression and anxiety, and thus patients may benefit from psychosocial therapy. Although preventing ICH is the primary concern with brain AVMs, the rest of the patient’s profile should not be forgotten.
